# Wastewater Treatment of Real Effluents by Microfiltration Using Poly(vinylidene fluoride–hexafluoropropylene) Membranes

**DOI:** 10.3390/polym15051143

**Published:** 2023-02-24

**Authors:** Djamila Zioui, Pedro Manuel Martins, Lamine Aoudjit, Hugo Salazar, Senentxu Lanceros-Méndez

**Affiliations:** 1 Unité de Développement des Équipements Solaires (UDES), Centre de Développement des Energies Renouvelables (CDER), Bou Ismail 42415, Algeria; 2 Centre of Molecular and Environmental Biology, University of Minho, Campus de Gualtar, 4710-057 Braga, Portugal; 3 Institute of Science and Innovation on Bio-Sustainability (IB-S), University of Minho, 4710-057 Braga, Portugal; 4 BCMaterials, Basque Centre for Materials, Applications and Nanostructures, UPV/EHU Science Park, 48940 Leioa, Spain; 5 IKERBASQUE, Basque Foundation for Science, 48013 Bilbao, Spain; 6 Physics Centre of Minho and Porto Universities (CF-UM-UP), University of Minho, 4710-057 Braga, Portugal; 7 Laboratory of Physics for Materials and Emergent Technologies (LapMET), University of Minho, 4710-057 Braga, Portugal

**Keywords:** heavy metals, multifunctional, organic matter, polymeric membrane, salinity, water treatment

## Abstract

Over the last decades, the growing contamination of wastewater, mainly caused by industrial processes, improper sewage, natural calamities, and a variety of anthropogenic activities, has caused an increase in water-borne diseases. Notably, industrial applications require careful consideration as they pose significant threats to human health and ecosystem biodiversity due to the production of persistent and complex contaminants. The present work reports on the development, characterization, and application of a poly (vinylidene fluoride—hexafluoropropylene) (PVDF-HFP) porous membrane for the remediation of a wide range of contaminants from wastewater withdrawn from industrial applications. The PVDF-HFP membrane showed a micrometric porous structure with thermal, chemical, and mechanical stability and a hydrophobic nature, leading to high permeability. The prepared membranes exhibited simultaneous activity on the removal of organic matter (total suspended and dissolved solids, TSS, and TDS, respectively), the mitigation of salinity in 50%, and the effective removal of some inorganic anions and heavy metals, achieving efficiencies around 60% for nickel, cadmium, and lead. The membrane proved to be a suitable approach for wastewater treatment, as it showed potential for the simultaneous remediation of a wide range of contaminants. Thus, the as-prepared PVDF-HFP membrane and the designed membrane reactor represent an efficient, straightforward, and low-cost alternative as a pretreatment step for continuous treatment processes for simultaneous organic and inorganic contaminants’ remediation in real industrial effluent sources.

## 1. Introduction

The scarcity of potable water is one of the most concerning health issues nowadays due to the increasing contamination of water bodies related to urbanization and industrialization growth [1]. According to the World Health Organization (WHO), more than 3 million people die each year from diseases caused by poor water quality, and more than 2 billion people are short of water for their needs and basic hygiene [2]. Thus, drinking and groundwater treatment is hugely needed to provide drinking water, one of the main Sustainable Development Goals defined by UNICEF. 

Wastewater from coking processes, livestock, industrial activities, or landfill leachate usually contains complex contaminant species with different natures and chemistry, from organic matter to inorganic compounds and heavy metals [3]. The main bottleneck of current remediation systems is the large number of specific stages required to remove each contaminant, leading to the implementation of expensive and large structures. Therefore, a multifunctional approach that could mitigate contaminants from different natures, especially in developing countries where sophisticated techniques are not available, is an urgent need [4]. The main challenge when remediating real wastewater, contaminated by several compounds, is how to reach the simultaneous removal of multiple contaminants using a single process [5]. To provide clean and safe water, different technologies, such as coagulation [6], oxidation [7], adsorption [1], anaerobic and aerobic digestion [8], membrane bioreactors [9], ozonation [10], and advanced oxidation processes [11] have been employed.

Membrane technology, an alternative to the above-mentioned traditional processes, has grabbed interest based on its high pollutant rejection rates, simple operation at room temperature, no phase change, selective permeability, low energy consumption, and low environmental impact [12]. In the past 40 years, membrane-based separation technologies have grown exponentially and become a prosperous business, accounting for USD 13.5 billion in 2019 and expected to reach USD 19.6 billion in 2025 [13]. The membrane separation process is based on a modular membrane to separate the target contaminants from feed water based on its opening pores. These membranes are classified according to their pore size: microfiltration (0.1–10 µm), ultrafiltration (1–100 nm), nanofiltration (0.1–10 nm), and reverse osmosis (<0.1 nm) [14]. Among the different membrane-related technologies, microfiltration and ultrafiltration are expected to share the market due to their wide application in different industries. Microfiltration is a low-pressure physical separation process that uses a semipermeable membrane to remove suspended solids from a liquid stream. Microfiltration membranes usually present pore dimensions in the range of 0.02–10 µm and typically operate under relatively low pressures: 0.02 to 0.5 MPa. It has been widely adopted in integrated membrane reactors for desalination [15] and heavy metals removal [16], as it allows the separation of large molecular weight compounds (suspended or colloidal elements) under low-pressure conditions. Microfiltration membranes are extensively implemented in wastewater treatment plants worldwide. It is reported that this separation method can efficiently remove organics from water with more than 98% of efficiency [17].

Polymeric membranes have been the most used materials for microfiltration purposes, providing significant benefits to their implementation: chemical and mechanical stability, physical and chemical tunability, flexibility, and the potential to target specific contaminant species [12]. The control over the micro to macroporous structure opens the perspective to design membranes with different properties for covering various needs in water remediation applications [18]. Different polymeric materials are currently being used for water treatment applications, such as poly (tetrafluoroethylene) [19], poly (ether sulfone) [20], polysulfone [21], and poly (vinylidene fluoride) (PVDF). The variety of polymers, which defines the chemical nature of the membrane, associated with multiple membrane geometries, makes it possible to manufacture membranes with different properties and that are capable of covering various needs in specific application areas. Herein, PVDF, and its copolymers, is one of the polymers that has received more attention concerning its outstanding properties. PVDF-HFP is attracting interest as a candidate for wastewater remediation due to its high mechanical strength, excellent chemical resistance, thermal stability, and high processability [22]. Chemical resistance and mechanical stability are critical, as most of the microfiltration applications discharge municipal waste or pretreatment for seawater desalination [13]. The implementation of PVDF-based membranes is as well all around the world, dominating the market. GE Zenon Zeeweed, GE Zenon 1500, Pall Siemens Memcor, Dow, Toray, and Hydranautics are some implemented membranes in the municipal water treatment market. These commercial membranes are currently applied as pretreatment in drinking water purification—reducing the chemical oxygen demand (COD) to 5 mg L^−1^ and the turbidity to 5 NTU, in municipal wastewater treatment—reducing the suspended solids (SS) to 15 mg L^−1^ and the turbidity to 5 NTU, in industrial wastewater treatment—reducing the COD to 15 mg L^−1^ and the turbidity to 3 NTU, and in seawater desalination—minimizing the SS to 10 mg L^−1^. The total treated by using PVDF membranes reaches 5,000,000 tons per day [13]. 

The suitability of a membrane to separate the intended contaminants is directly related to its processing method, as the method and its parameters will strongly affect the morphological, physicochemical, thermal, and mechanical properties, as well as the overall efficiency. Most of the membranes mentioned above are prepared by non-solvent-induced phase separation (NIPS) [23]. Recently, the thermal-induced phase separation (TIPS) method attracted significant attention due to the possibility of easily producing highly interconnected porous membranes. According to this method, the temperature at which the solvent will be evaporated is crucial to determine the final properties of the membrane. At room temperature, the slow evaporation of the solvent promotes a well-distributed and interconnected micrometric porous structure [24]. The processing of highly interconnected porous membranes and the control of their meso to microstructure increases their performance when exposed to complex water matrices [16]. When comparing the separation by size proposed by the NIPS membranes, the presence of interconnected pores can trap other contaminants and enhance the water treatment performance.

This work is focused on processing a porous PVDF-HFP membrane by using the TIPS method and its application in wastewater treatment by a microfiltration process. The employment of a distinct processing method will address the relation between it and the relevant challenge of the simultaneous removal of a wide range of contaminants from water bodies.

## 2. Materials and Methods

### 2.1. Materials

Poly (vinylidene fluoride—hexafluoropropylene) (PVDF-HFP), with an HFP content of 12 w.% and a molecular weight of 600,000 g mol^−1^, was supplied by Solvay. N, N-Dimethyl formamide (DMF) was purchased from Sigma-Aldrich, Madrid, Spain.

### 2.2. PVDF-HFP Membrane Preparation

As previously reported, the PVDF-HFP porous membranes were prepared by the solvent casting technique [4,25]. Briefly, a defined amount of PVDF-HFP was dissolved in DMF under magnetic stirring in a PVDF-HFP/DMF ratio of 15/85 *v/v*. Then, the solution was poured into a Petri dish, and the solvent was evaporated at room temperature for a few days. Samples with a typical average thickness of 115 µm were obtained.

### 2.3. Membrane Characterization

The morphology of the composite membranes was studied by scanning electron microscopy (SEM) through a NanoSEM—FEI Nova 200 (FEG/SEM, operated at an accelerating voltage of 10 kV. The samples were previously cryogenically fractured in liquid nitrogen and coated with a thin gold layer by magnetron sputtering (Fision Instruments, Polaron SC502) for 30 s at ≈20 mbar and ≈20 mA.

Fourier transformed infrared spectroscopy in the attenuated total reflectance mode (FTIR/ATR) was used to evaluate the vibrational modes of PVDF-HFP using a Jasco FT/IR-4100 apparatus. The measurements were performed from 4000 to 600 cm^−1^ with a resolution of 4 cm^−1^. The relative fraction of the β-phase of the membranes were calculated according to Equation (1):

(1) $$F\left(\beta \right)=\frac{A_{\beta }}{\left(K_{\beta }/K_{\alpha }\right)A_{\alpha }+A_{\beta }}$$

where *A_α_* and *A_β_* are the absorbance at 763, and 838 cm^−1^, respectively, and *K_α_* and *K_β_* (6.1 × 10^4^ and 7.7 × 10^4^ cm^2^ mol^−1^, respectively) are the absorption coefficients at the respective wavenumber.

Thermogravimetric analysis (TGA) was performed with a TGA/SDTA 851e Mettler Toledo apparatus under a high-purity nitrogen atmosphere (99.99% minimum purity) at a flow rate of 50 mL min^−1^. Samples of approximately 4 mg were placed in an aluminum oxide crucible and heated from 25 to 850 °C at a heating rate of 10 °C min^−1^.

Differential scanning calorimetry (DSC) was carried out under a nitrogen atmosphere with a Mettler Toledo DSC 822e, using 4 mg samples in aluminum pans. The measurements were conducted from 25 to 400 °C at a heating rate of 5 °C min^−1^.

Uniaxial stress-strain measurements evaluated the mechanical properties of the PVDF membrane were evaluated by uniaxial stress-strain measurements, using a Shimadzu AG-IS tests machine equipped with a 50 N load cell. The mechanical tests were performed at a 1 mm min^−1^ rate until the break, using samples with a 7 × 15 mm geometry and a thickness of ≈115 µm. Five replicates were performed.

Dead-end cell filtration experiments were made to access the membrane flux and permeability of the membrane and the implemented setup. Membrane flux (*J_W_*) and permeability (*k*) were calculated following Equations (2) and (3), respectively:

(2) $$J_{W}=\frac{\Delta V}{\Delta t\times A}$$

(3) $$k=\frac{J_{w}}{P_{TMP}}$$

where Δ*V* is the volume (L) of water, Δ*t* is the time (h), *A* is the area of the membrane (m^2^), and *P_TMP_* is the applied transmembrane pressure (kPa)

Contact angle measurements (sessile drop in dynamic mode) were performed at room temperature in a Data Physics OCA20 device using ultrapure water (3 µL droplets) as the test liquid. At least three measurements on each sample were performed in different sample locations, and the average contact angle was calculated.

The density of the samples (expressed as mass (mg) per volume (cm^3^)) was measured by weighing an area of the produced membranes and measuring the average thickness based on the SEM cross-section micrographs and using Image J software.

The water content (*W_content_*) was evaluated (Equation (4)) using a 20 × 20 mm sample, which was weighed in the dry state and subsequently immersed in distilled water at room temperature. After 24 h, the membranes were placed on absorbent paper to remove excess water and weighed.

(4) $$W_{content}=1-\frac{m_{d}}{m_{w}}\times 100$$

where *m_d_* is the mass of the dry membrane, and *m_w_* is the mass of the wet membrane.

The reproducibility of the membrane was evaluated by performing three experiments in the same conditions, followed by error analysis. The standard deviation (SD) and the relative standard deviation (RSD) were calculated following Equations (5) and (6), respectively [26]:

(5) $$SD=\sqrt{\frac{\sum \left(x_{i}-{\bar{x}}^{2}\right)}{n-1}}$$

(6) $$RSD=\frac{SD}{\bar{x}}\times 100$$

where *x_i_* is the experimentally obtained concentration in each replicate, $\bar{x}$ is the mean for each parameter, and *n* is the number of replicates.

### 2.4. Wastewater Characterization

The wastewater was obtained from a river inside an important industrial area near the Bouismail region—about 40 km southwest of Algiers. The relevance of this sample is related to its proximity to the sea. Samples were stored at 4 °C, transported to the laboratory, and kept in the fridge to avoid degradation until further pretreatments.

The samples’ pH, conductivity, and salinity were measured by a digital multiparameter (HANNA pH meter HS5221). The total suspended solids (TSS) and the total dissolved solids (TDS) were determined as shown in Equations (7) and (8), respectively, by filtering 500 mL of the samples through a 0.7 μm pore filter (weighted (KERN ABP 100-5DM ± 0.1 mg) *P*_0_; afterward, the filter was dried at 105 °C in an oven (Memmert UFE400) for two hours and then weighted *P*_2_). For the estimation of TDS, 200 mL of the filtrate was placed in a 250 mL porcelain crucible, previously weighted (*P*_1_), and then placed in the oven at 180 °C for 12 h. After cooling, the crucible was weighted (*P*_3_).

(7) $$TSS\;\left(mg/L\right)=\left(P_{2}-P_{0}\right)\times 2000$$

(8) $$TDS\;\left(g/L\right)=\left(P_{3}-P_{1}\right)\times 5$$

The total alkaline content (TAC), total hardness (TH), calcium (Ca^2+^), and chloride (Cl^−^) were measured by volumetric titration based on complexation. The concentration of cadmium, lead, zinc, and silver ions was determined at different time intervals by flame atomic absorption spectroscopy (flame–AAS) using a Perkin Elmer Analyst 700 spectrophotometer.

### 2.5. Filtration System

**Figure 1:**
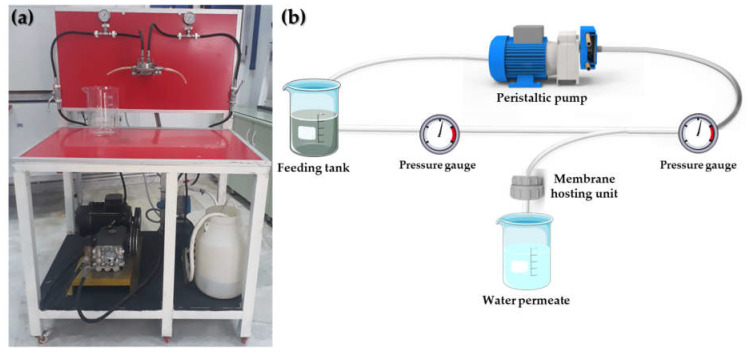
(**a**) Real picture and (**b**) schematic representation of the filtration setup.

The wastewater treatment was conducted using a lab-scale system (Figure 1a). The system consists of a filtration cell containing three outputs: feed, permeate, and concentrate, and an adjustable high-pressure pump (SIH high-pressure stainless-steel chemical pump). The setup is also composed of two adjusted valves (standard stainless steel) placed at the inlet and outlet of the cell (Figure 1b).

The wastewater treatment using the filtration setup was realized following optimized experimental parameters, which were previously studied to achieve the best performance: flow rate of 160 L h^−1^ m^−2^; pressure of 1 bar, and filtration time of 1 h. The water properties were measured before and after the filtration experiment to evaluate the performance of the membrane and setup. Three experiments were performed in the same conditions, and a 28.4 cm^2^ membrane was placed in the filtration cell for each one.

## 3. Results and Discussion

### 3.1. Membrane Characterization

**Figure 2:**
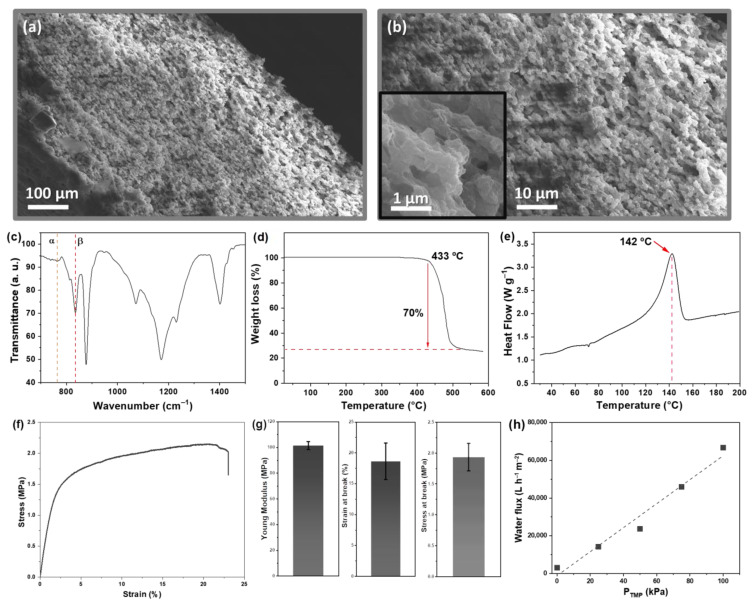
(**a**) Cross-section and (**b**) surface SEM micrographs; (**c**) FTIR-ATR spectra; (**d**) TGA; (**e**) DSC curves; (**f**) stress-strain curves; (**g**) mechanical properties; and (**h**) permeability of PVDF-HFP membrane.

SEM was used to assess the morphology and microstructure of the PVDF-HFP membranes. Figure 2a,b shows representative surface and cross-section images. The prepared membranes present a well-distributed micrometric and homogeneous pore structure along the cross-section and the top surface, promoted by a liquid–liquid phase separation process [4]. The PVDF-HFP porous morphology is composed of well-defined and interconnected pores with an average diameter of around 1–3 µm. The average thickness of the membranes is 115 µm. This homogeneity and interconnection of the pore structure are essential to achieve an adequate membrane water flux and permeability [27].

The FTIR spectra of the PVDF-HFP membranes Figure 2c presents the characteristic vibration modes of the α and β-phases of the polymer at 763 and 838 cm^−1^, respectively. The crystallization occurred mainly as a highly polar β-phase (71%), characteristic of PVDF-HFP membranes processed by a solvent casting technique and attributed to the low solvent evaporation temperature [28]. Moreover, characteristic absorption bands of C–F stretching, C–C symmetrical stretching, F–C–F, and C(F)–C(H)–C(F) skeletal bending are identified at 1401, 1171, 1072, and 878 cm^−1^.

The thermal properties of the membrane were studied by TGA (Figure 2d) and DSC (Figure 2e). From the TGA results, it is verified that the PVDF-HFP membrane decomposes in one step, starting at ~433 °C, confirming the excellent thermal stability of the membrane for the intended application. A mass loss of 70% is observed in this single degradation step. A DSC thermogram confirms the melting temperature (T_m_) of the crystalline phase of the copolymer at 142 °C, characteristic of PVDF-HFP [29].

The mechanical stress-strain curve of the PVDF-HFP membrane is shown in Figure 2f. The prepared membrane shows good mechanical strength, with a maximum strain above 20%. Compared with other porous structures obtained by different processing techniques, the lower maximum strain is related to the higher porosity of the herein-prepared membrane [30]. By performing mechanical experiments, it is possible to access some mechanical properties of the membrane (Figure 2g). The Young modulus obtained for the membrane is near 100 MPa, similar to other PVDF porous structures. A maximum strain of ≈20% and a maximum stress of ≈2.0 MPa can be applied to the membrane until the break, demonstrating its good mechanical properties.

According to Figure 2h, in a linear correlation, the membrane flux increases by increasing the transmembrane pressure. A permeability of 0.64 L m^−2^ h^−1^ Pa^−1^ was obtained for the PVDF membrane. Despite the hydrophobic nature of PVDF-HFP, the higher porosity of the herein-prepared membrane endows a significant permeability, which is helpful for water remediation applications [31].

Further, the PVDF-HFP membranes show a density of 44.2 mg cm^−3^ and a surface contact angle of 137°. In agreement with the wettability results, the 0% water content confirms the hydrophobic nature of the membranes [22].

### 3.2. Membrane Filtration

The main physical-chemical properties of the wastewater sample, before and after filtration by the PVDF-HFP membranes, and their comparison with the maximum concentration levels (MCLs) are presented in Table 1. The MCL is defined as the uppermost concentration of a particular compound that should be found in drinking water. The imposition of MCL values safeguards that drinking water presents no safety risks, as most MCLs are established between 10 and 10,000 below the amount that could cause health issues.

Implementing the membrane induces the separation of the contaminants at a high flow rate: 160 L h^−1^ m^−2^ bar^−1^. According to Table 1, the pH value of the wastewater sample after filtration is within acceptable limits and at a neutral value. The filtration system results in a significant decrease (50%) in the electrical conductivity of the wastewater, leading to conductivity values below the MCL. One of the main targets of the membrane and the filtration system was desalination, and a reduction of 39% in the salinity of the wastewater to values below the maximum allowed. A reduction in the TSS by 62.4% from 79.8 to 30.0 mg L^−1^ after the filtration was also observed, leading to values below the MCL. For the TDS, just a slight reduction was obtained (18%), which is related to the dimensions of the pores. From the volumetric titration of the permeate, the filtration process significantly decreases the hardness of the water, with a reduction of 99%.

**Figure 3:**
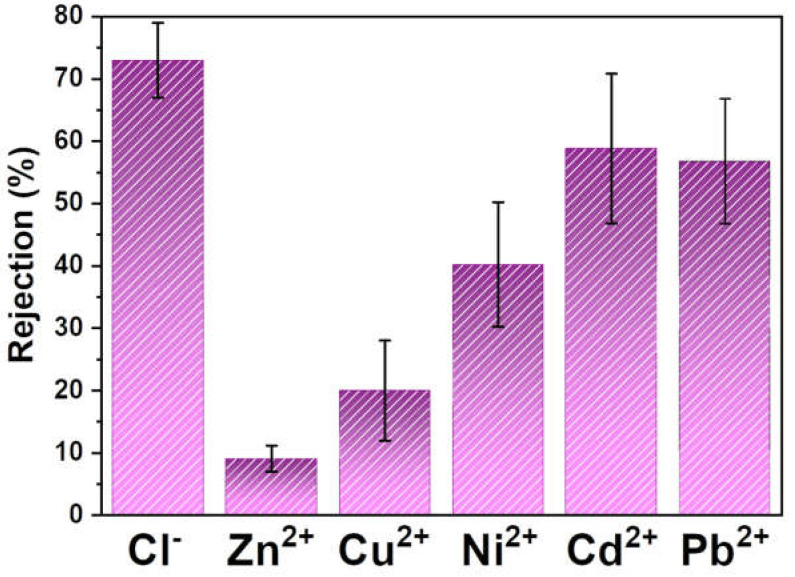
Performance of the PVDF-HFP membranes concerning the filtration of heavy metals and inorganic contaminants.

The removal of heavy metals and inorganic contaminants from wastewater was also evaluated under the same experimental conditions (Figure 3).

Directly related to the salinity results, the filtration of Cl^−^ ions were successfully performed by the PVDF-HFP membrane down to concentrations below the MCL. Regarding the filtration of inorganic cations, different efficiencies were obtained. Zn^2+^ and Cu^2+^ were unsuccessfully filtrated by the membrane, while Ni^2+^, Cd^2+^, and Pb^2+^ achieved significant removal efficiencies. As microfiltration is a separation technique based on the size of the elements in water, the larger elements would be more easily retained. Thus, the removal efficiencies can be explained by the ionic radius of each contaminant. Zn^2+^ and Cu^2+^ have the smallest ionic radius, followed by Ni^2+^, Cd^2+^, Pb^2+^, and then Cl^−^, according to the following tendency:

Cu^2+^ (73 pm) < Zn^2+^ (74) < Ni^2+^ (83) < Cd^2+^ (96) < Pb^2+^ (119) < Cl^−^ (181) [32,33].

As a result, Zn^2+^ and Cu^2+^ should percolate through the membrane pores easier and, therefore, are not significantly retained, while the larger ions (Cd^2+^, Pb^2+^, Cl^−^) are more effectively trapped within the polymeric matrix. With respect to the cations, the opposite charges between them and the fluorinated chains of PVDF-HFP may induce electrostatic interactions and promote their trap within the matrix [34]. The results indicate that the produced membranes can remove almost all inorganic contaminants until concentration levels below the MCLs.

A crucial parameter for evaluating the applicability of a membrane for water remediation is its reproducibility. To unravel this parameter, three experiments were performed under the same conditions, and the results can be discussed by error analysis of both Table 1 and Figure 3. The relative standard deviations (RSDs) are presented in Table 2.

Non-representative RSD values were found for the pH, conductivity, salinity, and organic content. It is fair to conclude the effectiveness and robustness of the PVDF membrane and the separation process for reducing organic content from wastewater, as similar efficiencies were achieved for the three experiments. With respect to inorganic anions and heavy metals, significant RSDs were obtained after the three experiments. As mentioned previously, the produced PVDF membrane can remove the contaminants through a microfiltration process. This separation process makes it possible to retain heavy metals and inorganic anions due to their small size. The increased removal efficiency also proves this aspect increased with the increased ionic radius of contaminants. One possible explanation for the significant retention of heavy metals is their association with the organic matter in the wastewater sample, leading to the agglomeration in larger molecules and favoring separation [35]. The higher RSD values could then be explained, as the separation efficiency depends on heavy metal–organic matter interactions.

## 4. Conclusions

Porous PVDF-HFP membranes were prepared by thermally induced phase separation for wastewater treatment by a microfiltration process. A porous structure was obtained with homogeneously distributed pores with an average diameter of around 1–3 µm. The membrane shows excellent chemical and thermal stability and hydrophobic nature. The membranes were applied for wastewater remediation by evaluating their performance for desalination and removal of organic and inorganic compounds (i.e., heavy metals), under a high flow rate. 

The desalination was successfully performed, as conductivity and salinity were above the MCL after the filtration process, presenting efficiencies of 50 and 39%, respectively. The organic matter filtration was evaluated in terms of TSS and TDS retention. Suspended solids were also effectively filtrated by the membrane, as shown by the 62.4% of efficiency, while dissolved solids seem to percolate through the membrane to the permeate. Furthermore, the effectiveness of the membrane for the removal of inorganic compounds and heavy metals was partially demonstrated, as significant removal efficiencies (55–75%) were achieved for Ni^2+^, Cd^2+^, and Pb^2+^, and the retention until levels below the MCL was obtained for Cl^−^.

In summary, the prepared PVDF-HFP membranes implemented in a membrane reactor proved to be a suitable approach to be implemented in wastewater treatment systems as a first treatment step, as their potential has been shown for the filtration of a wide range of contaminants—from desalination to organic and inorganic compounds, to heavy metals. Moreover, the low cost of this technique, related to its low applied pressure, encourages its implementation in up-scaled treatment systems.

## Figures and Tables

**Table 1:** Physical-chemical characterization of the wastewater sample before and after filtration.

Parameter	Before Treatment	After Treatment	MCL [31]
pH	7.9	7.2 ± 0.2	6.5–8.5
Conductivity (mS cm^−1^)	46.7	23.5 ± 1.1	25
Salinity (mg L^−1^)	312	190 ± 6	250
TSS * (mg L^−1^)	79.8	30.0 ± 2.1	40
TDS * (g L^−1^)	23.0	18.8 ± 1.5	5
TAC * (°F)	450	110 ± 6	-
TH * (°F)	9500	136 ± 10	-
Ca^2+^ (mg L^−1^)	561	200 ± 12	300
Cl^−^ (g L^−1^)	56.7	15.3 ± 1.7	5
Zn^2+^ (mg L^−1^)	0.33	0.30 ± 0.06	3
Cu^2+^ (mg L^−1^)	0.15	0.12 ± 0.03	0.5
Ni^2+^ (mg L^−1^)	0.92	0.55 ± 0.10	0.5
Cd^2+^ (mg L^−1^)	0.17	0.07 ± 0.02	0.2
Pb^2+^ (mg L^−1^)	1.32	0.57 ± 0.11	0.5

* TSS—total suspended solids; TDS—total dissolved solids; TAC—total alkaline content; TH—total hardness.

**Table 2:** Relative standard deviations for all the wastewater parameters—obtained by performing three experiments in the same conditions.

**RSD (%)**	**pH**	**Conductivity**	**Salinity**	**TSS**	**TDS**	**TAC**	**TH**
	2.7	4.7	3.1	7.0	8.0	5.5	7.3
**RSD (%)**	**Ca^2+^**	**Cl^−^**	**Zn^2+^**	**Cu^2+^**	**Ni^2+^**	**Cd^2+^**	**Pb^2+^**
	6.0	11	20	25	18	29	19
